# Comparison of Volume-Guaranteed or -Targeted, Pressure-Controlled Ventilation with Volume-Controlled Ventilation during Elective Surgery: A Systematic Review and Meta-Analysis

**DOI:** 10.3390/jcm10061276

**Published:** 2021-03-19

**Authors:** Volker Schick, Fabian Dusse, Ronny Eckardt, Steffen Kerkhoff, Simone Commotio, Jochen Hinkelbein, Alexander Mathes

**Affiliations:** Department of Anesthesiology and Intensive Care Medicine, University Hospital and Medical Faculty, Cologne University, Kerpener Str. 62, 50937 Cologne, Germany; volker.schick@uk-koeln.de (V.S.); fabian.dusse@uk-koeln.de (F.D.); ronny.eckardt@uk-koeln.de (R.E.); steffen.kerkhoff@uk-koeln.de (S.K.); simone.commotio@uk-koeln.de (S.C.); jochen.hinkelbein@uk-koeln.de (J.H.)

**Keywords:** volume controlled ventilation, pressure controlled ventilation, volume guarantee, volume target, auto-flow, PCV-VG, perioperative, surgery, anesthesia

## Abstract

For perioperative mechanical ventilation under general anesthesia, modern respirators aim at combining the benefits of pressure-controlled ventilation (PCV) and volume-controlled ventilation (VCV) in modes typically named “volume-guaranteed” or “volume-targeted” pressure-controlled ventilation (PCV-VG). This systematic review and meta-analysis tested the hypothesis that PCV-VG modes of ventilation could be beneficial in terms of improved airway pressures (P_peak_, P_plateau_, P_mean_), dynamic compliance (C_dyn_), or arterial blood gases (P_a_O_2_, P_a_CO_2_) in adults undergoing elective surgery under general anesthesia. Three major medical electronic databases were searched with predefined search strategies and publications were systematically evaluated according to the Cochrane Review Methods. Continuous variables were tested for mean differences using the inverse variance method and 95% confidence intervals (CI) were calculated. Based on the assumption that intervention effects across studies were not identical, a random effects model was chosen. Assessment for heterogeneity was performed with the χ^2^ test and the I^2^ statistic. As primary endpoints, P_peak_, P_plateau_, P_mean_, C_dyn_, P_a_O_2_, and P_a_CO_2_ were evaluated. Of the 725 publications identified, 17 finally met eligibility criteria, with a total of 929 patients recruited. Under supine two-lung ventilation, PCV-VG resulted in significantly reduced P_peak_ (15 studies) and P_plateau_ (9 studies) as well as higher C_dyn_ (9 studies), compared with VCV [random effects models; P_peak_: CI −3.26 to −1.47; *p* < 0.001; I^2^ = 82%; P_plateau_: −3.12 to −0.12; *p* = 0.03; I^2^ = 90%; C_dyn_: CI 3.42 to 8.65; *p* < 0.001; I^2^ = 90%]. For one-lung ventilation (8 studies), PCV-VG allowed for significantly lower P_peak_ and higher P_a_O_2_ compared with VCV. In Trendelenburg position (5 studies), this effect was significant for P_peak_ only. This systematic review and meta-analysis demonstrates that volume-targeting, pressure-controlled ventilation modes may provide benefits with respect to the improved airway dynamics in two- and one-lung ventilation, and improved oxygenation in one-lung ventilation in adults undergoing elective surgery.

## 1. Introduction

Mechanical ventilation is a common perioperative requirement, and modern respirators enable mechanical ventilation in a variety of modes [1]. New ventilation strategies aim at combining the advantages of two modes of ventilation, pressure-controlled ventilation (PCV) and volume-controlled ventilation (VCV). While pressure-controlled ventilation modes allow a decelerating flow, this technique may result in a relevant variation of the achieved tidal volume. VCV, on the other hand, more or less guarantees a set volume of ventilation, but uses a fixed flow, and this can result in variations of the achieved peak and/or plateau airway pressure. To combine both, modern ventilation modes aim at using a pressure-controlled way of applying flow and ventilation, with digital feedback mechanisms continuously controlling the applied tidal volume, targeting for a fixed tidal volume. The compliance of the lung is calculated by the ventilator to deliver the target tidal volume using the lowest possible pressure. These ventilation modes are called “volume guaranteed” (PCV-VG) or “volume targeted” pressure-controlled ventilation. Depending on the manufacturer, the names “dual control mode”, “auto-flow”, “Pressure Regulated Volume Controlled (PRVC) ventilation” or unique variations are also in use.

For the most common form of perioperative ventilation, i.e., continuous mandatory ventilation (CMV), there has been an ongoing debate whether PCV or VCV may offer advantages or disadvantages for the general patient or in certain subpopulations, for specific perioperative procedures (e.g., one-lung ventilation) or patient positioning (e.g., prone position, Trendelenburg position) [2,3]. Up to date, there is no consensus on whether PCV or VCV may significantly improve the patient outcome in the perioperative setting [4,5]. 

For laparoscopy and the Trendelenburg position, research showed potential advantages for PCV-VG in the perioperative setting, e.g., in terms of improved oxygenation and reduced airway pressures [6]. However, other publications did not show an improved oxygenation for this new ventilation mode [7], and Sahutoglu even described a non-significant trend toward the more post-operative complications after the use of PCV-VG [8]. Other comparisons of PCV-VG in the perioperative setting, e.g., with PCV itself, are sparse and limited to three studies up to date. To shed more light on this new form of perioperative ventilation, we therefore hypothesized that pressure-controlled, volume-guaranteed ventilation modes (PCV-VG) may have advantages over conventional, volume-controlled ventilation (VCV) during surgery with respect to airway pressures and blood gas analysis. Therefore, we conducted a systematic review and meta-analysis, comparing ventilation with PCV-VG and VCV in adults undergoing elective surgery. 

## 2. Materials and Methods

A systematic review and meta-analysis were performed according to the PRISMA guidelines [9] and the Cochrane Handbook for Systematic Reviews of Interventions [10]. This systematic review was not registered in international databases prior to the investigation.

### 2.1. Search Strategy, Elegibility Criteria, and Study Selection

NCBI/PubMed (including MEDLINE), Cochrane and EMBASE were searched for eligible studies using the general search strategy given in Table 1. Each strategy was modified for the technical interface of the database and included all relevant fields (e.g., title, abstract and keywords) as well as all word variations (e.g., with and without hyphens) according to the instructions for the algorithm of the database. All searches were performed in an open, non-truncated fashion and included automatic term mapping functions as well as thesaurus functions, according to each database. In addition, manual searches included secondary references and citations of the relevant literature to find potentially unlisted publications. 

In- and exclusion criteria were based on the PICOS model [9,10] and defined as follows. Inclusion Criteria: Participants = adult patients ≥ 18 years; Interventions = elective surgery; Comparisons = pressure-controlled ventilation with a volume-guaranteed or volume targeted mode (PCV-VG) versus volume-controlled ventilation (VCV); Outcomes = blood gas analysis, respirator mechanics and airway pressures as primary endpoints, hemodynamic variables and adverse events as secondary endpoints; Study design = randomized controlled trials. Exclusion Criteria: Participants = patients < 18 years, children, neonates; Interventions = non-elective surgery, e.g., emergency surgery, critical care ventilation; Comparisons = missing comparison or other modes of ventilation; Outcomes = case reports or interventions not reporting outcomes, or outcomes not appropriate for meta-analysis; Study design = not randomized trials, not full studies, e.g., case reports or abstracts. Potential subgroups were defined ex ante as positioning (supine, prone, Trendelenburg) and one-lung ventilation based on a primary survey of the literature.

Three reviewers (V.S., R.E., and S.C.) independently evaluated all search results using the program AbstrackR^®^ (Center for Evidence Synthesis in Health; Brown University; Providence, RI; USA) in a blinded fashion based on the abovementioned criteria. An additional, independent referee (J.H.) dissolved discrepancies regarding the question whether a study should be included. 

### 2.2. Data Extraction

Two reviewers (F.D. and A.M.) independently extracted the data from the included studies; again, an additional, independent referee (J.H.) dissolved discrepancies regarding the question whether or how a set of data should be included and confirmed data extraction. Data extraction was based on the predefined outcome criteria. The following variables were collected: number of patients (*n*); mode of ventilation; tidal volume; reference for tidal volume; airway pressures P_peak_, P_plateau_, P_mean_, dynamic compliance C_dyn_, positive end expiratory pressure (PEEP); blood gas analysis: pH, p_a_O_2_, p_a_CO_2_; hemodynamic variables: heart rate, mean arterial blood pressure (MAP), central venous pressure (CVP); patient positioning; type of surgery; reporting of adverse events.

### 2.3. Study Quality and Risk of Bias

Two reviewers (F.D. and A.M.) independently evaluated the risk of bias for each publication using the Cochrane Risk of Bias tool version 2 (RoB-2; Centre for Research Synthesis and Decision Analysis; University of Bristol; Bristol; UK) [11]; results were graphically plotted using the Risk of Bias visualization software Robvis (Bristol Appraisal and Review of Research Group; University of Bristol; Bristol; UK) [12]. Again, an additional, independent referee (J.H.) dissolved discrepancies regarding this evaluation.

### 2.4. Statistical Analysis

The parameters outlined above were obtained from the included studies at the given time points. For publications stating interquartile ranges and medians (*n* = 3), standard deviations were estimated using the formula described in Section 6.5.2.5. of the Cochrane Handbook for interquartile ranges [10]. For publications stating median, minimum, and maximum values (*n* = 1), means and standard deviations were estimated using the method described by Hozo [13]. 

All statistical analyses were performed with RevMan version 5.4 (The Cochrane Collaboration; London, UK). Continuous variables were tested for mean differences using the inverse variance method and 95% confidence intervals (CI) were calculated. Based on the assumption that intervention effects across studies were not identical, a random effects model was chosen [14]. Assessment for heterogeneity was performed with the χ^2^ test and the I^2^ statistic. An I^2^ > 50% and a χ^2^ test with *p* < 0.10 were considered to indicate statistical heterogeneity; in absence of asymmetry, the random effects model was applied. In publications with a cross-over design, only a single set of data prior to cross-over was used, and all further cross-over data were excluded from this meta-analysis, to eliminate the risk of bias by carry-over effects.

## 3. Results

### 3.1. Study Selection

**Figure 1:**
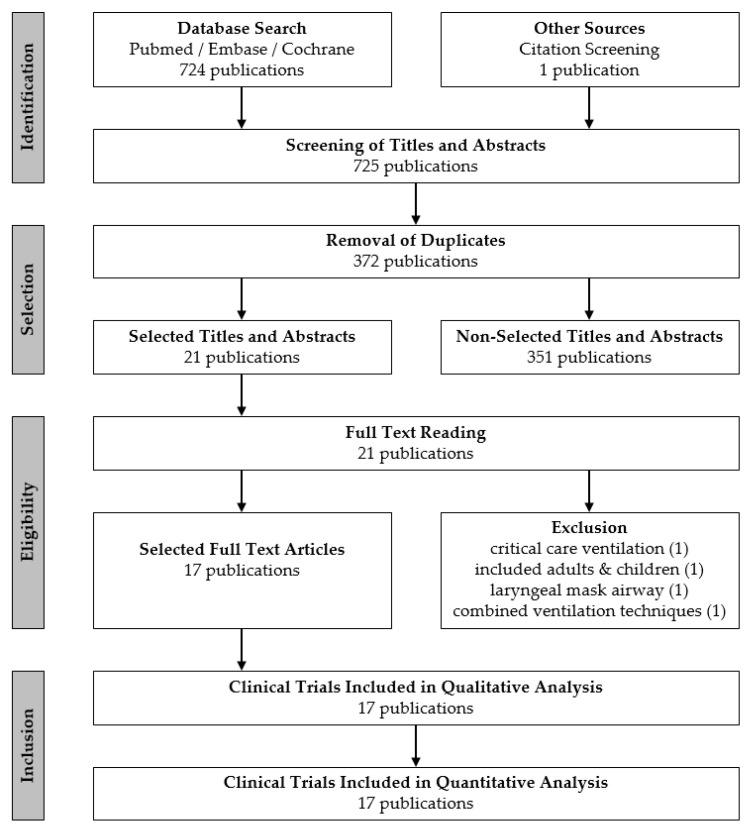
Flow chart of study selection.

Initially, 724 publications in NCBI/PubMed, Embase, and Cochrane were identified. Additional records and manual search resulted in one study, for a total of 725 publications. After duplicates were removed, title and abstract of 372 publications were screened for selection according to the PICOS criteria specified above. Of those, 351 had to be excluded, resulting in 21 publications that were full-text assessed for eligibility. Four publications had to be excluded, finally resulting in 17 studies that underwent the following qualitative evaluation [6,7,8,15,16,17,18,19,20,21,22,23,24,25,26,27,28]. A flow chart of the search strategy and study selection are shown in Figure 1.

### 3.2. Study Characteristics

Within 17 publications included for this meta-analysis, a total of 929 patients were enrolled (average: 55 patients per investigation; range: 20–100 patients). Almost 60% of all investigations (*n* = 10) were published within the last three years (2018–2020), and 94% of all studies (*n* = 16) were published within the last 7 years (2014–2020); no study was older than 10 years. Over 60% of the studies were performed in Asian countries (*n* = 11). There was no investigation from North or South America, Australia, Great Britain, or an EU country. For study characteristics, please see Table 2.

### 3.3. Study Quality and Risk of Bias

**Figure 2:**
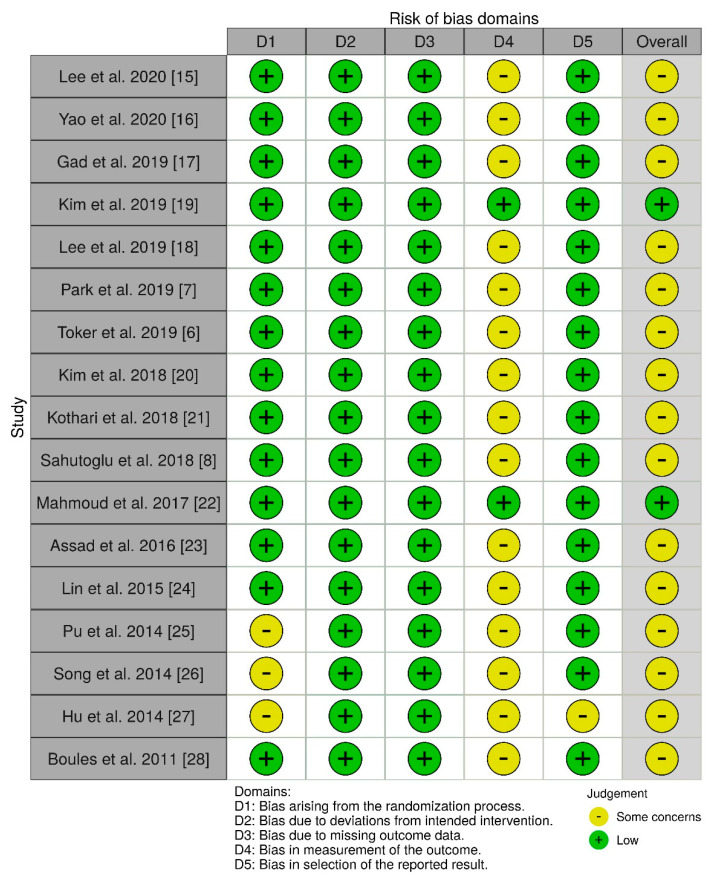
Risk of bias traffic light plot.

**Figure 3:**
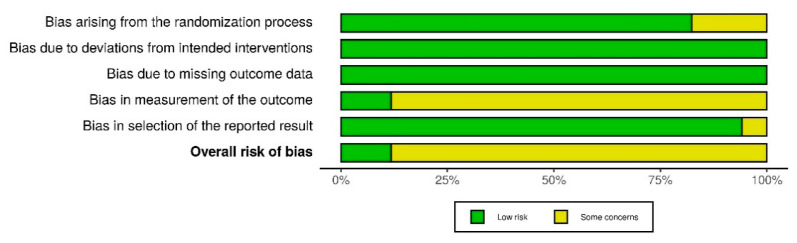
Risk of bias summary plot.

All of the 17 included studies used a randomized, controlled design and were approved by the local/institutional review board. Most of the publications were of moderate to high quality. In general, allocation processes were randomized and patients were unaware of their treatment; baseline differences were not detected and there was no missing data. In many studies, it was either not clear how the investigator (e.g., the anesthesiologist performing the ventilation) was blinded with respect to the ventilation mode, or the study was explicitly not blinded in this regard. However, according to the Cochrane Risk of Bias methods, even if outcome assessors were aware of the intervention and if assessment of the outcome may—in theory—have been influenced by the knowledge of intervention received, it remains highly unlikely that assessment of the outcome was in fact influenced by the knowledge of the intervention received, as most endpoints were measured using objective, validated, or calibrated devices (e.g., airway pressures or blood gas analysis). Accordingly, risk of detection bias is only rated “high” for endpoints that are vulnerable to subjective evaluation (like non-calibrated treatment effects or subjective symptoms of patients), but not regarding measurements by calibrated devices. Therefore, the risk of detection bias (D4) was only moderate in this meta-analysis. Yet, for the same reason, risk of bias assessing adherence to intervention resulted in “some concerns” for a number of investigations included in this meta-analysis (Figure 2 and Figure 3).

### 3.4. Statistical Heterogeneity

All included endpoints (P_peak_, C_dyn_, p_a_O_2_, and p_a_CO_2_) showed a significant heterogeneity by either I^2^ > 50%, a χ^2^ with a *p* < 0.10, or both. In the absence of relevant asymmetry, the random effects model was applied to all analyses. 

### 3.5. Airway Pressures 

Of the 17 studies included, 15 reported peak airway pressure (P_peak_), 9 studies reported plateau airway pressures (P_plateau_), and 13 studies reported mean airway pressures (P_mean_) for supine two-lung ventilation without capnoperitoneum. Data for dynamic airway compliance (C_dyn_) were available for nine studies. For all endpoints, data sets were chosen from the earliest time point under general anesthesia in supine position with sufficient equilibration for two-lung ventilation (e.g., 5–10 min after induction, 10 min in supine position or after 5–30 min of two-lung ventilation).

**Figure 4:**
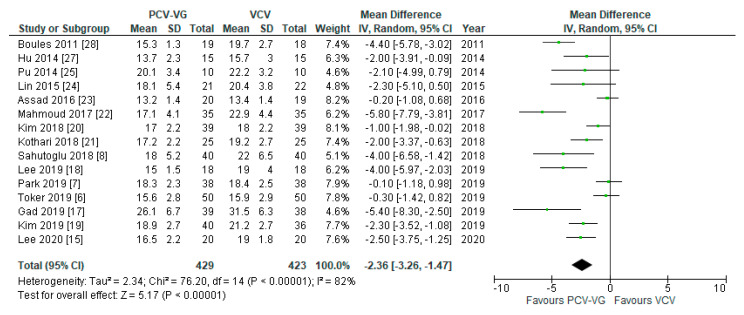
Peak airway pressure (P_peak_): supine, two-lung ventilation. The green center of each square represents the weighted mean difference for individual trials, and the corresponding horizontal line stands for a 95% confidence interval. The diamonds represent pooled results.

**Figure 5:**
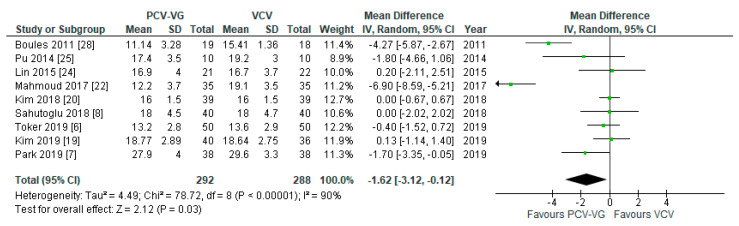
Plateau airway pressure (P_plateau_): supine, two-lung ventilation. The green center of each square represents the weighted mean difference for individual trials, and the corresponding horizontal line stands for a 95% confidence interval. The diamonds represent pooled results.

**Figure 6:**
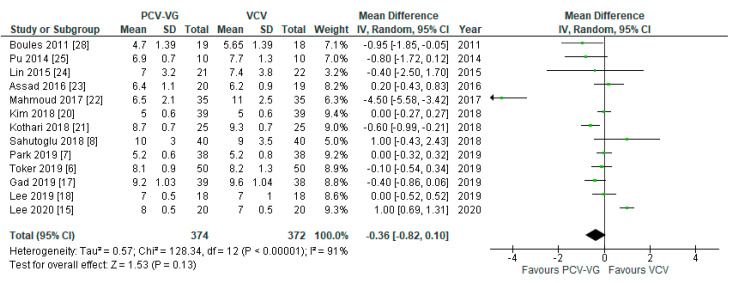
Mean airway pressure (P_mean_): supine, two-lung ventilation. The green center of each square represents the weighted mean difference for individual trials, and the corresponding horizontal line stands for a 95% confidence interval. The diamonds represent pooled results.

**Figure 7:**
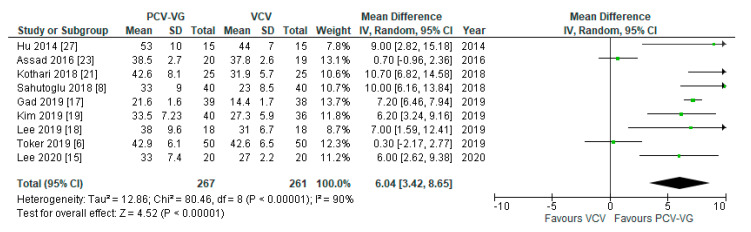
Dynamic compliance (C_dyn_): supine, two-lung ventilation. The green center of each square represents the weighted mean difference for individual trials, and the corresponding horizontal line stands for a 95% confidence interval. The diamonds represent pooled results.

Peak airway pressures were significantly lower in patients ventilated with PCV-VG undergoing supine two-lung ventilation, compared with VCV under the same conditions (*p* < 0.00001; Figure 4); the same result was found for P_plateau_ (*p* < 0.03; Figure 5). P_mean_ was not different between both ventilation modes (*p* = 0.13; Figure 6). In addition, dynamic compliance was significantly higher within the same comparison (Figure 7). 

### 3.6. Blood Gas Analysis

**Figure 8:**
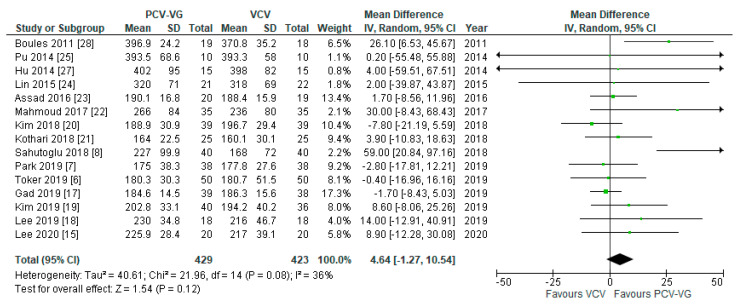
Arterial oxygen pressure (P_a_O_2_): supine, two-lung ventilation. The green center of each square represents the weighted mean difference for individual trials, and the corresponding horizontal line stands for a 95% confidence interval. The diamonds represent pooled results.

**Figure 9:**
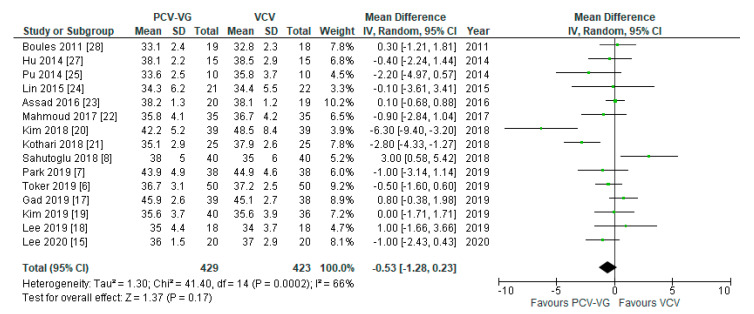
Arterial carbon dioxide pressure (P_a_CO_2_): supine, two-lung ventilation. The green center of each square represents the weighted mean difference for individual trials, and the corresponding horizontal line stands for a 95% confidence interval. The diamonds represent pooled results.

Of all the included studies, 15 reported arterial oxygen pressure (P_a_O_2_) and arterial carbon dioxide pressure (P_a_CO_2_) for supine two-lung ventilation without capnoperitoneum. There was no significant difference between both ventilation modes with respect to arterial oxygen (*p* = 0.12; Figure 8) and carbon dioxide (*p* = 0.17; Figure 9) levels.

### 3.7. Subgroup Evaluation: One-Lung Ventilation

**Figure 10:**
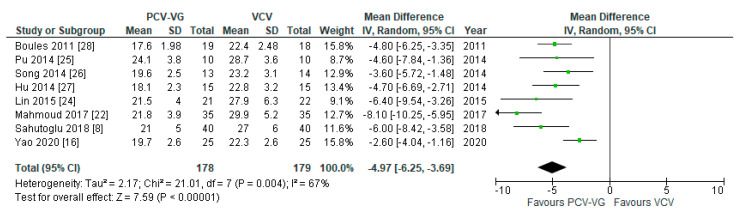
Peak airway pressure (P_peak_): after 20–60 min of one-lung ventilation. The green center of each square represents the weighted mean difference for individual trials, and the corresponding horizontal line stands for a 95% confidence interval. The diamonds represent pooled results.

**Figure 11:**
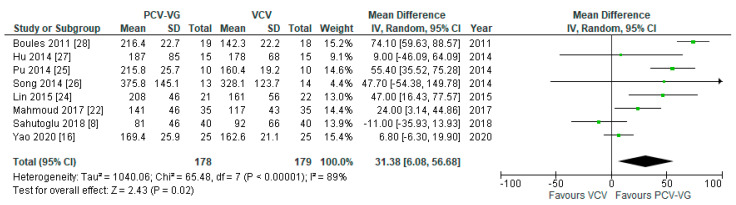
Arterial oxygen pressure (P_a_O_2_): after 20–60 min of one-lung ventilation. The green center of each square represents the weighted mean difference for individual trials, and the corresponding horizontal line stands for a 95% confidence interval. The diamonds represent pooled results.

Eight publications reported results for P_peak_, P_a_O_2_, and P_a_CO_2_ after 20 min (*n* = 1), 30 min (*n* = 3), and after 60 min (*n* = 4) of one-lung ventilation. As this set of data is different from the above with respect to time of measurement and patient position, these data are presented in a separate analysis below, and not included as a true subgroup. Peak airway pressures (*p* < 0.00001; Figure 10) were significantly lower and arterial oxygen pressures (*p* = 0.02; Figure 11) were significantly higher in patients ventilated with PCV-VG undergoing one-lung ventilation, compared with VCV under the same conditions. There was no significant difference between both ventilation modes with respect to arterial carbon dioxide levels (*p* = 0.67; Forest plot not shown).

### 3.8. Subgroup Evaluation: Trendelenburg Position

**Figure 12:**
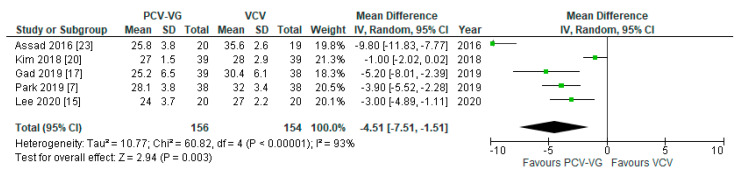
Peak airway pressure (P_peak_): after 60 min of Trendelenburg position. The green center of each square represents the weighted mean difference for individual trials, and the corresponding horizontal line stands for a 95% confidence interval. The diamonds represent pooled results.

Five investigations presented various parameters for variable time points of Trendelenburg positions. In all studies, P_peak_ was reported for 60 min of Trendelenburg positioning. P_peak_ was significantly lower in patients ventilated with PCV-VG after 60 min in Trendelenburg position, compared with VCV under the same conditions (*p* = 0.003; Figure 12). There was no significant difference between both ventilation modes with respect to the arterial oxygen and carbon dioxide levels (P_a_O_2_: *p* = 0.94; P_a_CO_2_: *p* = 0.61; Forest plots not shown).

### 3.9. Hemodynamic Data 

Hemodynamic parameters were reported only inconsistently or inhomogeneous and sometimes incomplete, and were therefore not evaluated further. In general, all investigators reported stable hemodynamic conditions for all patients. Further, no adverse events were detected with respect to the hemodynamic instability (see below).

### 3.10. Adverse Events and Additional Outcome Parameters

In eight publications, adverse events and various postoperative outcome parameters were explicitly reported (Table 3). In all other studies, those endpoints were not mentioned. Whenever postoperative events were investigated, most parameters did not show any significant differences between groups. Park et al. reported significantly more postoperative fever in VCV-ventilated patients [7]. Mahmoud and colleagues reported significantly improved outcomes, like postoperative oxygenation and even a reduced ICU and hospital stay, in PCV-VG-ventilated patients compared with VCV [22]. Yao and colleagues observed significantly lower neutrophil elastase levels after PCV-VG ventilation compared with VCV [16]. Sahutoglu et al. reported a non-significant trend toward more complications in PCV-VG, however, individual complications were almost identical between groups [8]. In general, no serious adverse events were reported.

## 4. Discussion

This systematic review and meta-analysis included 17 randomized, controlled trials that compared PCV-VG with VCV in 929 patients scheduled for elective major non-cardiac surgery under general anesthesia. PCV-VG aims at combining the advantages of VCV and PCV to ensure a target tidal volume with a decelerating flow. Depending on the previous breath, lung compliance is being calculated and the tidal volume is applied with the lowest inspiratory pressure possible [1,29]. So far, several studies have examined the effects of PCV-VG compared with the various conventional modes of ventilation, like VCV. However, the published data gave no clear direction for or against the superiority of any specific ventilation technology. Therefore, in this meta-analysis, we investigated the potential short-term outcome parameters e.g., airway compliance, airway pressures, and gas exchange in PCV-VG compared to the conventional, volume-controlled ventilation. Of the 17 included studies, 15 reported peak airway pressure, arterial oxygen pressure, and arterial carbon dioxide pressure for supine two-lung ventilation. Nine studies presented data for plateau airway pressure and dynamic airway compliance, and five gave data for a specific period of Trendelenburg position.

As this meta-analysis shows, peak airway and plateau pressures were significantly lower in patients ventilated with PCV-VG undergoing supine two-lung ventilation, compared with VCV. Regarding these endpoints, only three studies showed no difference for peak airway pressures, while the other twelve demonstrated moderately to significantly reduced peak airway pressures when using PCV-VG. These findings are in line with the previous meta-analyses comparing VCV and PCV—yet without volume targeting (“PCV-non-VG”)—resulting in significantly lower peak inspiratory pressures when using a PCV-non-VG mode, compared with VCV [5,30]. The decelerating airway flow, usually characterizing pressure-controlled ventilation, and digital feedback mechanisms may contribute further to the reduction of the peak airway pressure using PCV-VG. 

High airway pressures and large tidal volumes during mechanical ventilation are not part of a lung protective ventilation strategy and may therefore cause acute lung injury [31]. As our study shows that PCV-VG was associated with lower P_peak_ and lower P_plateau_ than similar ventilation with VCV, this could have the potential to correspond with a decreased risk of ventilator-induced lung injury. This idea is supported by the (single) report on a significantly reduced neutrophil elastase after the use of PCV-VG [16], as well as the finding that total complications, ICU and hospital stay were significantly reduced in the PCV-VG group [22]. On the other hand, this is not in line with the study of Sahutoglu, indicating a non-significant trend toward more postoperative complications in the PCV-VG group [8]. Yet, this meta-analysis was not designed to evaluate the potential effects on lung injury, and therefore, these interpretations can only be preliminary.

The clinical relevance of peak airway pressure for the development of barotrauma—especially in the course of intraoperative ventilation—is controversial. Some authors propose that peak airway pressure could not properly reflect the alveolar pressure [5,32]. On the other hand, peak airway pressure is the most frequently reported value, and one of the most important, clinically most useful, and readily available parameter regarding airway pressures in ventilation practice. Most importantly, it was the most common parameter comparing the two ventilation strategies across all included studies. 

Evaluation and interpretation of long-term effects using PCV-VG or VCV is challenging. Multiple postoperative outcome parameters were mentioned in eight out of the 17 publications. Only one study reported a significant difference for postoperative outcome parameters with reduced ICU and hospital stay in the PCV-VG group [22]. Yet, while questions on long-term outcomes may not be satisfyingly answered by this evaluation, the reported data on adverse events—although limited—also allow the assumption that PCV-VG does not result in an increased number of serious adverse events, compared with VCV.

No significant differences were seen for arterial oxygen pressure between VCV and PCV-VG. However, only four out of 15 included studies showed mild to moderate reductions in arterial oxygenation in the PCV-VG group, compared with the VCV group. Therefore, based on the reported data, serious disadvantages in respect of arterial oxygenation using PCV-VG cannot be postulated. In the same fashion, no significant differences were seen for arterial carbon dioxide pressure for both ventilation techniques. 

Data for airway compliance were extracted from nine studies. All of those included studies showed an improvement in compliances using PCV-VG compared to VCV. Accordingly, our analysis reveals significant superiority of the PCV-VG technique with respect to dynamic airway compliance as a functional lung parameter in mechanical ventilation. 

A subgroup analysis including nine studies for one-lung ventilation was carried out with regard to the parameters peak airway pressure, arterial oxygen pressure, and arterial carbon dioxide pressure. Comparable results for peak airway pressure were found, but a significantly higher arterial oxygen pressure was measured using PCV-VG. There were no significant differences in oxygen saturation. Changes in pressure conditions during one-lung ventilation, different measurement time, and patient positioning, however, were potential confounders that may blur these results. Most of the studies had excluded patients with poor preoperative pulmonary function or severe lung diseases. Assuming that this particularly critically ill patient group would benefit from PCV-VG, the effect of PCV-VG might have been underestimated. The mechanism for this beneficial effect of PCV-VG on oxygenation during one-lung ventilation remains to be established. It is tempting to speculate that PCV-VG might have an impact on the closing capacity during thoracic surgery. This could—in theory—be caused by the fact that the flow during PCV-VG is not fixed, potentially resulting in a higher ventilation of small airways in the environment of a higher extraluminal pressure. 

This meta-analysis has various limitations. First, PCV-VG is a relatively new mode of ventilation, and only 17 investigations fulfilled the inclusion criteria for our meta-analysis. Especially in subgroup analyses, a relatively small number of patients was included and the range of patients per study was 20–100, indicating that intervention effects could be significantly overstated in small study groups. 

Second, double blinding was not performed by all investigators due to the chosen study design and clinical needs. As discussed above, this should not have had a relevant impact on the results, as measurements of airway pressures and blood gases were performed using calibrated machines. Therefore, these data were not prone to subjective falsification. In accordance with the Cochrane Methods, the risk of bias due to this effect was consequentially only moderate, and we believe that the data are reliable. 

Third, all studies derived from selected regions of the world. This may theoretically have an impact on our findings, as loco-regional effects of a health-care system cannot be excluded completely. On the other hand, the objective measurement of the primary endpoints of this meta-analysis should not have been influenced by the origin of the studies.

Fourth, regarding all primary endpoints as well as subgroup analysis for one-lung ventilation, measurements for airway pressures and blood gas analysis were taken at different time points (e.g., 20, 40, or 60 min after one-lung ventilation). Therefore, for interpretation of the results, individual factors of each study member and surgical progress must be taken into account. 

Fifth, although data were generally symmetrical, most evaluations revealed a relevant heterogeneity of the studies included. To draw substantial conclusions for clinical practice, large-scale investigations with rigorous inclusion criteria would be needed. Due to the heterogeneity observed, the results need to be interpreted with caution.

Finally, it was not possible to extract uniform long-term parameters as these were not reported regularly. Therefore, long-term effects due to different intraoperative ventilation strategies (e.g., VCV or PCV-VG) cannot be assessed. However, this was not a primary endpoint of this investigation.

Yet, this meta-analysis has also certain strengths: to our knowledge, this is the first systematic review of PCV-VG compared with VCV. Further, this investigation includes the data of over 900 patients from 17 prospective, randomized trials. Over 90% of the data is relatively new (2014+) and derives from relatively homogenous populations. All endpoints were obtained using strictly objective measurements with a very low risk of bias. Consequently, overall risk of bias in all studies was low to moderate and intervention effects were, to the best of our knowledge, properly estimated. Further, measurements for mechanical ventilation and gas exchange have been well reported. All recorded endpoints were related to the intraoperative or direct postoperative period. Therefore, this meta-analysis—despite some limitations—may add a significant amount to our knowledge on intraoperative ventilation regarding this new volume-targeting ventilation mode, PCV-VG. 

Our investigation raises an important clinical question: Based on our findings, should this new mode of ventilation, PCV-VG, be routinely used in the perioperative setting? From a historical perspective, many modern ventilation modes have failed to allow for significant, long-term improvements of clinical practice. Yet, PCV-VG has the potential to combine two former “opponents” of ventilation in a single mode of action. As Ball and colleagues have put it into words, PCV-VG appears to offer “undoubted clinical advantages” [1]. Whether this mode will fulfill our high expectations for the perioperative setting, remains to be determined by future studies. Until these data are available, PCV-VG may not necessarily qualify as the “new standard” in the operating theatre, but could nonetheless prove to be useful for the individual patient.

## 5. Conclusions

This systematic review and meta-analysis indicate that PCV-VG may provide benefits in terms of improved airway dynamics compared to VCV in adults undergoing elective non-cardiac surgery. PCV-VG appears to be a safe ventilation technique without relevant disadvantages or even inferiority, with respect to the evaluated endpoints. In two lung-ventilation in supine position, peak airway and plateau pressure were significantly lower and dynamic compliance was significantly higher for PCV-VG-ventilated patients. In one-lung ventilation, peak airway pressures and oxygenation were improved. Overall, the use of the PCV-VG technique for intraoperative ventilation appears to be beneficial, although valid data of long-term outcome parameters for distinct ventilation modes remain to be established. 

## Figures and Tables

**Table 1:** Search strategies.

String	Condition	Search ^1^
#1	-	[‘ventilation’ OR ‘ventilator’ OR ‘respiration’ OR ‘respirator’]
#2	AND	[‘volume guarant*’ OR ‘volume-guarant*’ OR ‘volume target*’ OR ‘volume-target*’]
#3	OR	[‘autoflow’ OR ‘dual control mode’ OR ‘PCV-VG’ OR ‘PRVC’]
#4	AND	[‘perioperative’ OR ‘operating room’ OR ‘anesthesia’]
#5	NOT	[‘infant*’ OR ‘newborn’ OR ‘pediat*’ OR ‘child*’]
#6	NOT	[‘review’ OR ‘animal’]
#7	-	[#1 AND [#2 OR #3] AND #4] NOT #5 NOT 6

^1^ All word variations (*) and field searches according to the technical interface of the database.

**Table 2:** Study characteristics.

Publication	Year	Origin	Surgery	Anesthesia	Airway	Position	Tidal Volume	PEEP	I:E	*n*
Lee et al. [15]	2020	Korea	Robotic/LSC/OBS	balanced	ITN	45° TDB	8 mL/kg IBW	N/A	1:2	40
Yao et al. [16]	2020	China	TSC/Lobectomy	balanced	DLT	N/A	6–10 mL/kg	5	1:2	50
Gad et al. [17]	2019	Egypt	LSC/HE	balanced	ITN	30° TDB	6–8 mL/kg	0	1:2	77
Lee et al. [18]	2019	Korea	lumbar spine	balanced	ITN	prone	8 mL/kg IBW	N/A	1:2	36
Park et al. [7]	2019	Korea	Robotic/LSC/PSE	balanced	ITN	Steep TDB	8 mL/kg IBW	0	1:2	76
Toker et al. [6]	2019	Turkey	LSC/HE	balanced	ITN	30° TDB	8 mL/kg PBW	5	1:2	100
Kim et al. [19]	2019	Korea	hip joint	balanced	ITN	LD	8 mL/kg	5	1:2	76
Kim et al. [20]	2018	Korea	Robotic/LSC/PSE	balanced	ITN	30° TDB	8 mL/kg IBW	0	1:2	78
Kothari et al. [21]	2018	India	LSC/CHE	balanced	ITN	Head Up	8 mL/kg	5	1:2	50
Sahutoglu et al. [8]	2018	Turkey	Lobectomy	balanced	DLT	Lateral	4–7 mL/kg PBW	4	1:2	80
Mahmoud et al. [22]	2017	Egypt	Thoracotomy	TIVA	DLT	Lateral	6–10 mL/kg	10	1:2	70
Assad et al. [23]	2016	Egypt	LSC	balanced	ITN	TDB	8 mL/kg	0	1:2	39
Lin et al. [24]	2015	China	Thoracic	TIVA	DLT	Lateral	8–10 mL/kg	N/A	1:2	43
Pu et al. [25]	2014	China	Thoracic	TIVA	DLT	N/A	8–10 mL/kg	N/A	1:2	20
Song et al. [26]	2014	Korea	Thoracic	TIVA	DLT	Lateral	8 mL/kg ABW	0	1:2	27
Hu et al. [27]	2014	China	TSC/Lobectomy	balanced	ITN	Lateral	7 mL/kg	N/A	1:1.5	30
Boules et al. [28]	2011	Egypt	Thoracic	balanced	DLT	Lateral	6–10 mL/kg IBW	0	1:2	37

ABW = actual body weight; CHE = cholecystectomy; DLT = double lumen tube; HE = hysterectomy; IBW = ideal body weight; I:E = inspiratory:expiratory ratio; ITN = intubation; LSC = Laparoscopic; N/A = not available; OBS = obstetric; PBW = predicted body weight; PEEP = positive end expiratory pressure; PSE = prostatectomy; TDB = Trendelenburg; TIVA = total intravenous anesthesia; TSC = Thoracoscopic.

**Table 3:** Adverse events and additional outcome parameters.

Publication	Adverse Event	PCV-VG	VCV	*p*-Value	Report on Adverse Events
Yao 2020 [16]	PACU time	1.79 h	1.56 h	0.150	No difference between groups
	Re-intubation	4%	4%	0.750	NE significantly lower in PCV-VG
	Lung infection	8%	4%	0.500	
	Hospital stay	7.5 d	8.2 d	0.100	
Park 2019 [7]	PostOP fever	3%	12%	**0.022**	Significantly more post-OP fever in VCV
Kim 2019 [19]	-	-	-	-	No critical complications
Kim 2018 [20]	PostOP fever	20.5%	28.2%	0.429	No difference between groups
	PACU time	48 min	45 min	0.813	
	Hospital stay	3 d	2 d	0.275	
	30d-readmission	7.7%	7.7%	0.999	
Sahutoglu 2018 [8]	> 1 complication	25%	7.5%	0.066	No difference for individual complications
Mahmoud 2017 [22]	Total complications	14%	46%	**0.004**	Significantly shorter ICU and hospital stay and less complications in PCV-VG
	Pneumonia	9%	17%	0.284	
	ARDS	0%	6%	0.151	
	Atelectasis	6%	17%	0.133	
	ICU stay	19.2 h	29.1 h	**0.013**	
	Hospital stay	8.1 d	13.2 d	**0.033**	
Assad 2016 [23]	-	-	-	-	Reporting of no complications, but only 1 h post-OP observation time
Boules 2011 [28]	Basal atelectasis	3 pat	4 pat	n.s.	No significant difference in post-OP lung expansion

Bold values indicate significant findings. ARDS = adult respiratory distress syndrome; NE = neutrophil elastase; ICU = intensive care unit; OI = oxygenation index; PACU = post anesthesia care unit; PCV-VG = pressure-controlled ventilation with volume guarantee/targeting; VCV = volume-controlled ventilation.

## Data Availability

No new data were created or analyzed in this study. Data sharing is not applicable to this article.
